# Distracted Driving Behavior Among Adults in the Perambalur District: A Cross-Sectional Study

**DOI:** 10.7759/cureus.40864

**Published:** 2023-06-23

**Authors:** Tamilarasan Muniyapillai, Karthikeyan Kulothungan, Maniprabhu S, Harini Meera

**Affiliations:** 1 Community Medicine, Dhanalakshmi Srinivasan Medical College and Hospital, Perambalur, IND; 2 Community Medicine, K.A.P. Viswanatham Government Medical College, Trichy, IND

**Keywords:** india, road accidents, road traffic accidents, total distracted driving scale, roadway hazards, distracted driving

## Abstract

Background

Distracted driving is a major public health concern. Distraction results in reduced speed control, lateral lane position, reduced situational awareness, and impaired response times to roadway hazards. Visual, cognitive, and manual distractions impair drivers in different ways. With the above background, this study was conducted with the objective of estimating the proportion of distracted driving behavior and its predictors among the adult population in the Perambalur district, Tamil Nadu, India, by using the Total Distracted Driving Scale.

Materials and methods

A cross-sectional study was conducted among 403 adults aged 18 years and above by convenience sampling technique in the Perambalur district for six months. A pretested, semi-structured proforma was used to collect data on socio-demographic characteristics such as age, sex, education, and occupation. To quantify distracted driving behavior, the Total Distracted Driving Scale was used. This scale contains seven questions about talking on the phone, five questions about texting, and five questions about using specific devices while driving. Data were entered into Microsoft Excel and analyzed using the Statistical Package for the Social Sciences (SPSS) Version 21. Descriptive statistics were used to describe the distracted driving behavior with respect to each variable, and to find out the significance, a corresponding statistical test was employed. A p-value of less than 0.05 was considered statistically significant.

Results

The mean age of the study participants was 24.86 ± 10.63 years. More than half of the study subjects (52.4 percent) were females, and around 87.3 percent of them were Hindu by religion. Among the study participants, around 66.74 percent mentioned that they had used a cell phone while driving. Around 38 percent of the study participants admitted that they had been in or were near-crash circumstances during the past year. Around 87 percent of the study participants who had a crash or near-crash in the past year admitted that they had been distracted while driving. On primary analysis, religion of individuals and increased driving frequency had a statistically significant association with a history of distracted driving. Study participants who were using three- and four-wheelers and those who used cellphones while driving had a statistically significant association with a history of distracted driving. According to the regression, cell phone users are 3.915 times more distracted than those who do not use cellphones (p = 0.001). Subjects with a history of crashes or near-crashes were 56.96 times more distracted than those without a history (p < 0.001).

Conclusion

In the present study, three-fourths of the study population used a cell phone while driving. More than three-fourths of the study participants admitted that they had been in a crash or near-crash circumstances during the past year. Distracted driving was responsible for four-fifths of all crashes or near-collisions. Use of a cell phone while driving and a history of near-crashes have a higher risk of distraction than those who do not.

## Introduction

Road traffic accidents (RTAs) are the eighth most common cause of death worldwide and the number one killer of children and young adults [1]. More than half of all RTA-related deaths occur to vulnerable road users (non-motorized road users, such as pedestrians and cyclists, as well as motorcyclists and persons with disabilities or reduced mobility and orientation) because frequently they are not shielded during collisions [2,3]. Alarmingly, 85 to 90 percent of these RTA deaths take place in middle-income countries (MIC), such as India, leaving behind young, productive age groups that suffer significant economic hardship and disability-adjusted life-years [2]. The majority of motorized two- and three-wheeler riders die in the South-East Asia region, and head injuries are the leading cause of mortality among them [1]. In India, RTA caused approximately 154,732 fatalities and 439,262 injuries in 2019, illustrating the severity of such terrible accidents [4].

Indian roadways have become perilous for commuters due to the trend of population increase, urbanization, industrialization, and a high rate of motorization combined with fast-moving automobiles. RTAs are believed to be caused by a number of factors, including human error in the form of traffic rule violations such as speeding, driving under the influence of alcohol or drugs, or driving while distracted, as well as poor road infrastructure, the threat of stray animals, automobiles lacking safety mechanisms, and inadequate regulation implementation [5,6].

Adolescent drivers account for a sizable portion of motor vehicle crashes and cases because they are more likely to be distracted while driving. Behind such situations, a wide variety of reasons are investigated. High usage of electronic devices, novice drivers, peer participation while driving, and risk-taking inclinations are just a few of the dangers that make young drivers more susceptible to accidents [7].

Time is the most important aspect of our lives, and thus we attempt to conserve as much of it as we can. Multitasking is necessary in this process to expedite our duties. People used to strive to multitask everything, but that reduced their ability to focus on each task individually. There is a chance that the task will fail if attention is not given. The same phenomenon happens while driving; individuals tend to eat, drink, and use their phones, which reduces their concentration on the road and increases the likelihood of accidents [8].

With the above background, the current study was conducted with the aim of assessing the study subjects according to distracted driving during a crash or near-crash that happened within the past year.

## Materials and methods

Study design and duration

We conducted a cross-sectional, analytical study for a duration of six months (April to September 2022).

Study population

We included the adult population older than 18 years in the Perambalur district, Tamil Nadu, India.

Inclusion criteria

Adults over the age of 18 years who were residents of those three villages in the Perambalur district and gave their willing consent were allowed to participate in the study.

Exclusion criteria

We excluded adult people who were not present in the residence even after three visits to the house on different days and at various times.

Sampling technique and sample size

By using a random number table, we selected three villages out of the 152 villages (including rural and urban) in the Perambalur district. After that, using a convenient sampling technique, we took all the adult population in those villages as study subjects. The principal investigator contacted them at their residence (all houses in the village). Individuals who were not present at the time of the visit were visited three times on different days and times. A study conducted by Gershon et al. in 2017 found that the prevalence of distracted driving was 58 percent [9]. With the above prevalence rate, we calculated the minimum sample size needed for this study by using the formula 3.84*p*q/d2, where p is prevalence, q is the complement of p, and d is precision (which is 5 percent absolute error). The minimum sample required for our study was 400.

Ethics committee clearance and informed consent

Before conducting the study, we obtained an ethics clearance certificate from the Institutional Ethics Committee of Dhanalakshmi Srinivasan Medical College and Hospital (approval number: IECHS/IRCHS/No.162). We explained the objectives of the study to participants before they gave their written consent to participate.

Data collection

We collected the data from the study subjects through face-to-face interviews with them. We used a pretested, semi-structured questionnaire to collect the data. The questionnaire had three parts. The first part consists of questions about their socio-demographic characteristics such as age, sex, education, occupation, and place of residence. The socio-economic status of the study participants was assessed using the modified B.G. Prasad scale [10]. The second part of the questionnaire consists of questions from the Total Distracted Driving Scale to quantify distracted driving behavior among the study participants [11]. This scale contains 17 questions. Of those, seven questions were about talking on the phone, five were about texting, and five were about using specific devices while driving. The third part contains questions about the history of crashes or near-crash incidents in the past year and the reason for distracted driving at the time of a crash or near-crash. The first question required a yes or no response. For the remaining 16 questions, respondents estimated their frequency of distracted driving using a Likert scale with response options ranging from "never" to "> 75 percent."

Crashes were defined as situations in which a study subject’s vehicle contacted any object, at any speed, including other vehicles, people, bicycles, animals, trees, or buildings. Non-premeditated departures from the roadway were also included if at least one tire left the road's paved or intended travel surface. Any situation that required a quick evasive action by the study participant's vehicle or any other vehicle, pedestrian, bicycle, or animal to escape a collision was referred to as a near-crash [12].

Statistical analysis

All the data we collected were entered into Microsoft Excel, and the SPSS Version 21 (IBM Corp., Armonk, NY) was used to analyze the results. The categorical variables were expressed as tables (as frequency and percentage) and a pie diagram. The quantitative variables were expressed in tables as mean and standard deviation. The association between the presence of distracted driving behavior among the study participants and the categorical predictor variables was assessed using the chi-square test/Fisher's exact test. Similarly, the association between the presence of distracted driving behavior and the quantitative predictor variables was performed using an independent t-test. Binary logistic regression was performed with the primary analysis variables that are statistically significant (p < 0.05). With a 95 percent confidence interval, a p-value of less than 0.05 was considered statistically significant.

## Results

We included 403 study subjects. The data collected from them were used for statistical analysis. The typical characteristics of the study population are described in Table 1. The mean age (± standard deviation) of the study participants was 24.86 ± 10.63 years. More than half of the study subjects (52.4 percent) were female, and around 87.3 percent of them were Hindu by religion. Around four-fifths of them reside in urban locales. More than half of the study participants belonged to classes 4 and 5 of the socio-economic class according to the modified B.G. Prasad scale. Most of the study participants in this study drove two-wheelers, which was 76.4 percent.

**Table 1:** General characteristics of the study population (n = 403) #Mean ± standard deviation

Variable		Frequency	Percentage
Age in years		24.86 ± 10.63^#^	
Gender	Male	192	47.6
	Female	211	52.4
Religion	Hindu	352	87.3
	Christian	35	8.7
	Muslim	16	4.0
Place of residence	Urban	323	80.1
	Rural	80	19.9
Socio-economic status	Class 1	38	9.4
	Class 2	48	11.9
	Class 3	92	22.8
	Class 4	128	31.8
	Class 5	97	24.1
Frequency of driving	Almost every day	137	34.0
	Few days a month	134	33.3
	Few days a week	81	20.1
	Few days a year	51	12.7
Type of vehicle	Two-wheelers	308	76.4
	Three-wheelers	3	0.7
	Four-wheelers	92	22.8

Table 2 lists the responses that the study participants provided to the total of 17 questions on the Total Distracted Driving Scale. Among the study participants, around 66.74 percent admitted that they had used a cell phone while driving. Around 40 out of 403 people admitted that they have used a portable music player while driving more than 75 percent of the time. Similarly, 13 people admitted that they were using their mobile phones for texting within city limits. Almost 35 study participants used a Global Positioning System (GPS) device while driving more than 75 percent of the time.

**Table 2:** Responses provided to the total of seventeen questions on the Total Distracted Driving Scale #Frequency and percentage of cell phone use while driving

Questions	Never	<10%	11-25%	26-50%	51-74%	>75%
Do you use a cell phone while driving? (either handheld or hands-free)	269 (66.74%)^ #^					
If you use a cell phone while driving, what percent of the time do you use a hands-free device?	251	72	32	18	7	23
While driving, what percent of the time do you talk on the phone in city streets (stopped at a light)?	207	118	31	15	21	11
While driving, what percent of the time do you talk on the phone in city streets (normal speed)?	250	80	36	16	18	3
While driving, what percent of the time do you talk on the phone in freeway driving (stop-and-go traffic)?	247	88	30	14	15	9
While driving, what percent of the time do you talk on the phone in freeway driving (normal speed)?	260	79	31	17	14	2
What percent of the time do you talk on the phone while driving on the freeway at a normal speed?	260	80	33	13	17	0
While driving, what percent of the time do you send text messages in city streets (stopped at a light)?	274	69	27	14	6	13
While driving, what percent of the time do you send text messages in city streets (normal speed)?	308	55	18	12	8	2
While driving, what percent of the time do you send text messages in freeway driving (stop-and-go traffic)?	296	53	28	9	13	4
While driving, what percent of the time do you send text messages in freeway driving (normal speed)?	312	57	13	10	9	2
While driving, what percent of the time do you send text messages when stopped at a red light?	263	66	29	15	9	21
While driving, what percent of the time do you use a GPS device?	204	90	43	14	17	35
While driving, what percent of the time do you use a portable music player?	224	73	32	18	16	40
While driving, what percent of the time do you use a laptop/tablet?	365	22	7	8	1	0
While driving, what percent of the time do you use a smartphone application?	310	67	14	8	4	0
While driving, what percent of the time do you use other electronic devices?	320	60	14	7	2	0

**Figure 1:**
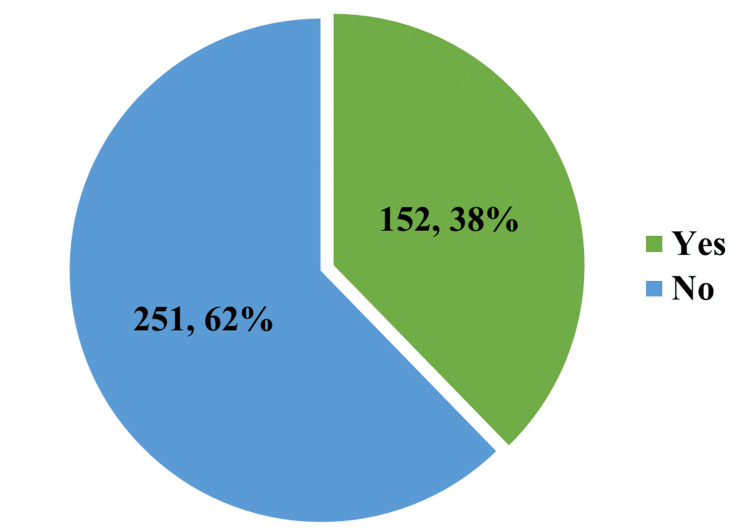
Percentage of crash or near-crash in the past one year among respondents (n = 403)

Figure 1 depicts the prevalence of a crash or near-crash in the previous year among study participants. Around 38 percent of the study participants admitted that they had been in crash or near-crash circumstances during the past year.

**Figure 2:**
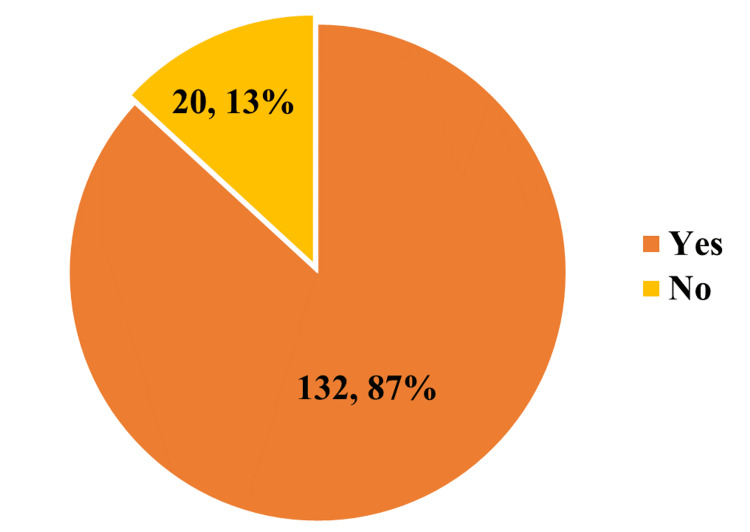
History of distraction at the time of the last crash or near-crash (n = 152)

Figure 2 depicts the prevalence of a positive history of disregarded driving among study participants during the most recent crash or near-crash. Around 87 percent of the study participants who had a crash or near-crash situation in the past year admitted that they had been distracted while driving.

Table 3 lists the justifications for distractions given by study participants during a crash or near-crash that occurred the previous year. Most of the participants (20 percent) do not know what the distraction was. Around 18 percent of them admitted daydreaming, and 16 percent admitted that they were talking to the passengers. Almost 13 percent of the study subjects were adjusting their radio or GPS during the crash. One-fifth of the participants were reading text messages on their phones, and around 8 percent of them were talking on the phone.

**Table 3:** Reason for distraction at the time of crash or near-crash (n = 132) GPS, Global Positioning System

Reason for distraction	Frequency	Percentage
Adjusting radio, GPS, etc.	17	13
Daydreaming	23	18
Eating	3	2
Grooming	10	8
Reading electronic text	8	6
Sending text message or e-mail	13	10
Talking to passengers	21	15
Talking on cell phone	10	8
Do not know	27	20

The association between typical characteristics and the history of distracted driving is shown in Table 4. According to Fisher's exact test, religion of individuals had a statistically significant history of distracted driving. Similarly, if the frequency of driving increases, the risk of getting distracted while driving also increases. This association was statistically significant according to the chi-square test. According to Fisher's exact test, study participants who were using three- and four-wheelers had a statistically significant association with a history of distracted driving. Individuals who were using a cell phone while driving had a statistically significant association with a history of distracted driving, according to the chi-square test.

**Table 4:** Predictors of distracted driving behavior among adults *Independent t-test was used. #Fisher's exact test was used.

Variables		History of distracted driving				P-value
		Yes		No		
		n	%	n	%	
Age^*^		25.70±11.617		24.45±10.118		0.270
Gender	Male	65	33.9	127	66.1	0.672
	Female	67	31.8	144	68.2	
Religion	Hindu	109	31.0	243	69	0.019^#^
	Christian	19	54.3	16	45.47	
	Muslim	4	25.0	12	75	
Place of residence	Urban	110	34.1	213	65.9	0.289
	Rural	22	27.5	58	72.5	
Socio-economic status	Class 1	9	23.7	29	76.3	0.122
	Class 2	11	22.9	37	77.1	
	Class 3	38	41.3	54	58.7	
	Class 4	45	35.2	83	64.8	
	Class 5	29	29.9	68	70.1	
Frequency of driving	Almost every day	50	36.5	87	63.5	0.077
	Few days a month	43	32.1	91	67.9	
	Few days a week	30	37.0	51	63	
	Few days a year	9	17.6	42	82.4	
Type of vehicle	Two-wheelers	89	28.9	219	71.1	0.001^#^
	Three-wheelers	3	100.0	0	0	
	Four-wheelers	40	43.5	52	56.5	
Cell phone use	Yes	113	42	156	58	0.0001
	No	19	14.2	115	85.8	

Binary logistic regression was performed with the following primary analysis variables that are statistically significant (p < 0.05): religion, frequency of driving, type of vehicle, cell phone use, and history of crashes or near-crashes. The regression model was significant (Wald value = 45.928, chi-square value = 258.629, p < 0.001) with cell phone use and history of crashes or near-crashes. According to the regression, cell phone users are 3.915 times more distracted than those who do not use cellphones (p = 0.001). Subjects with a history of crashes or near-crashes were 56.96 times more distracted than those without a history (p < 0.001).

## Discussion

We conducted this study with the aim of evaluating the study subjects according to distracted driving during a crash or near-crash that happened within the past year. Our results found that the prevalence of crashes or near-crash events in the past year among the study population was 38 percent. The prevalence of distracted driving among those who had a crash or near-crash was 87 percent. Among those who were distracted, the most common distractions were daydreaming and talking to the passengers. Thirteen percent of the participants claimed that they were adjusting their GPS, and around 10 percent admitted that they were using their mobile phones for texting. Our study also discovered that driving frequency and the use of three- and four-wheelers are significant risk factors for becoming distracted while driving, which could result in a crash or near-crash.

Similar to our study, a study conducted in the Emirates in 2020 by Alketbi et al. found that around 39.8 percent of the young drivers met with road traffic collisions [13]. In contrast to our study results, a study conducted by Hill et al. in 2014 among college students in California found that the most common distraction among drivers was texting while driving, which was 90 percent. According to the findings of the same study, there were 66 percent of women among them [11]. In our study, more than half of the participants were female. This difference in gender might be due to Indian culture's male preference for driving. The difference in the common distraction factor could be due to the subjective nature of the questions on the scale.

A study conducted by Neuroth et al. in 2021 among 62 young drivers in the USA found most study participants using cellphones while driving. Especially, around 43 percent of them said they use their cellphones for reading and texting messages. They also found that the young drivers had a positive perception of their ability to drive, even with mobile phone use. They also recommend inventing hands-free devices, which might be a better option for young drivers [14]. One more exploratory study was conducted by Delgado et al. in 2018 among teenage drivers with the aim of exploring the willingness to avoid cellphone use while driving. They conclude that most of the drivers are willing to avoid texting but are not willing to avoid mobile phone navigation or listen to music [15]. This discussion needs further exploration.

In addition to the above discussion, a qualitative study conducted by Watters and Beck in 2016 among 25 college undergraduates in the USA found that the most common distracted driving factor was texting while driving. They also conclude that this factor could possibly be the most difficult to stop among the adult population due to perceived barriers [16]. A review article by Khan et al. in India also concluded that the major distraction factor in the Indian adult population was the use of electronic gadgets [7]. This fact was challenged by our study, which found that talking to passengers and daydreaming were common reasons for distraction. They also conclude that most of the accidents were due to distracted driving, which was a similar finding when compared to our study.

In supporting our evidence, a study conducted by Majgi and AiswaryaLakshmi in Karnataka, India, found that the proportion of hand-held mobile phone users was 1.41 out of 100 vehicles. They also conclude that the proportion of drivers in India using mobile phones while driving is relatively low, especially on busy roads [17]. Our study opened a new path that required further research.

Limitations

Due to resource constraints, the current study took advantage of convenient sampling in one district. A multi-centric study with cluster sampling all over India could yield better results. Though the current study used a pre-validated questionnaire for evaluating distracted driving, all the questions were subjective in nature. Thus, it might create information bias or recall bias, which could influence the study results. Because of increased government awareness of the dangers of using mobile phones while driving, the prevalence of using a mobile phone while driving found in our study might be underestimated. Our study only focused on distracted driving during a crash or near-crash. We did not consider road infrastructure or traffic complexity for crashes or near-crash situations, which might influence the study results. The association found in this study might not be causal since this was a cross-sectional study.

## Conclusions

In the present study, three-fourths of the study population used a cell phone while driving. More than three-fourths of the study participants admitted that they had been in or were near-crash circumstances during the past year. Distracted driving was responsible for four-fifths of all crashes or near-collisions. Use of a cell phone while driving and a history of near-crashes have a higher risk of distraction than those who do not. Common distraction found in the Indian population during driving was daydreaming and talking to passengers. These reasons dominate the electronic gadget use in India. We recommend increasing awareness and behavior change communication among the adult population for distracted driving to improve individual personal responsibility during driving on Indian roads, which may reduce RTAs.
